# Penile Entrapment With Thick Penile Ring: A Case Report and Literature Review

**DOI:** 10.7759/cureus.21034

**Published:** 2022-01-08

**Authors:** Mohamed Zeid, Hani Sayedin, Nauman Nabi, Mamoun Abdelrahman, Prem T Jacob

**Affiliations:** 1 Urology, University Hospital Limerick, Limerick, IRL; 2 Urology, Warrington and Halton Teaching Hospitals NHS Foundation Trust, Warrington, GBR

**Keywords:** scrotal injury, penile strangulation, genitourinary trauma, penile ring entrapment, penile injury

## Abstract

The penis is one of the end-artery organs in the human body. The blood supply of the penis depends on the internal pudendal artery, which arises from the anterior division of the internal iliac artery. Subsequently, the penis is one of the organs that are highly affected by peripheral vascular disease. Furthermore, erectile dysfunction is a clinical sign that might precede coronary heart disease. Artificial entrapment of the blood into the cavernous bodies is one of the treatment options for erectile dysfunction. In addition, the same concept might be utilized in some sex aids to increase self-pleasure; hence, penile rings are widely used in some cultures. We present here a case of metal penile ring entrapment, which was managed successfully with the help of the hospital maintenance team. Therefore, it is of tremendous importance in unusual cases to seek advice from all possible resources. Such complications should be highlighted to increase the awareness of the users and the medical professionals as well.

## Introduction

Sex aids, also known as sex toys, refer to tools or devices that are used mainly to facilitate human sexual pleasure. The use of sex aids for more than 30,000 years has been reported by archeologists [1]. Moreover, one of the first treatment options for erectile dysfunction (ED) was detailed around 300 AD, detailing penis extensions made of wood or reeds tied to the waist. Constriction bands might be used to apply pressure at the base of the penile shaft resulting in obstruction of venous blood outflow, helping to increase penile rigidity and to improve the penile erection [2]. Given the fact they are non-invasive, constrictor bands proved to be effective in the management of ED, climacturia, and orgasm-associated incontinence as well. Mehta et al. reported about 50% improvement in the severity of climacturia and the degree of bother in patients who had radical prostatectomy [3]. Hence, the use of penile rings as a sex aid might be popular in some cultures, particularly where they are available. Penile ring entrapment during sexual self-satisfaction might be one of the rare conditions in urological surgery. When the shaft of the penis becomes trapped in a metal ring, it causes a series of vascular events including venous stasis, worsening edema, reduced lymphatic and arterial flow, and eventual gangrene [4].

## Case presentation

**Figure 1:**
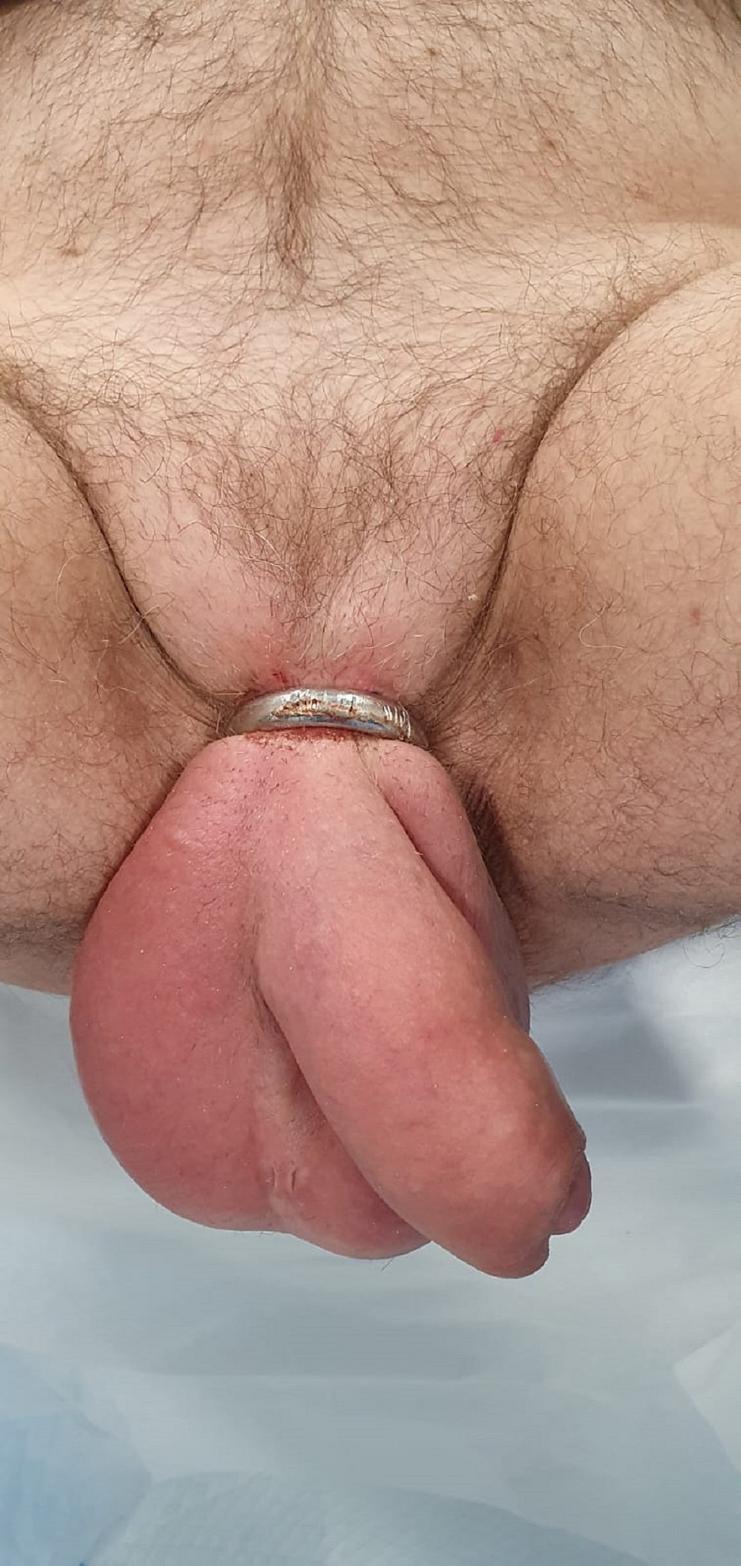
Penile ring entrapping both the penis and the scrotum (dorsal view).

**Figure 2:**
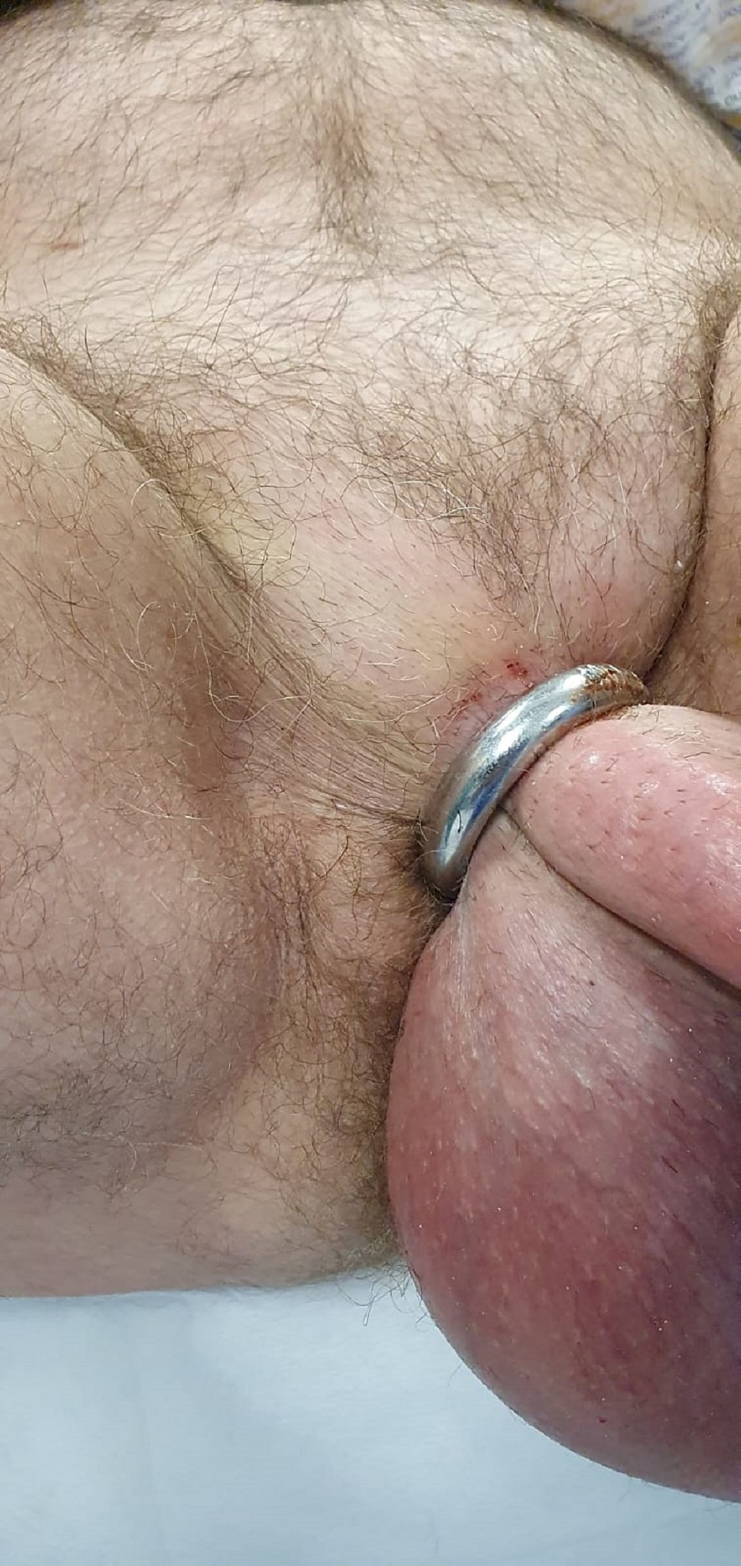
Penile ring entrapping both the penis and the scrotum (side view).

**Figure 3:**
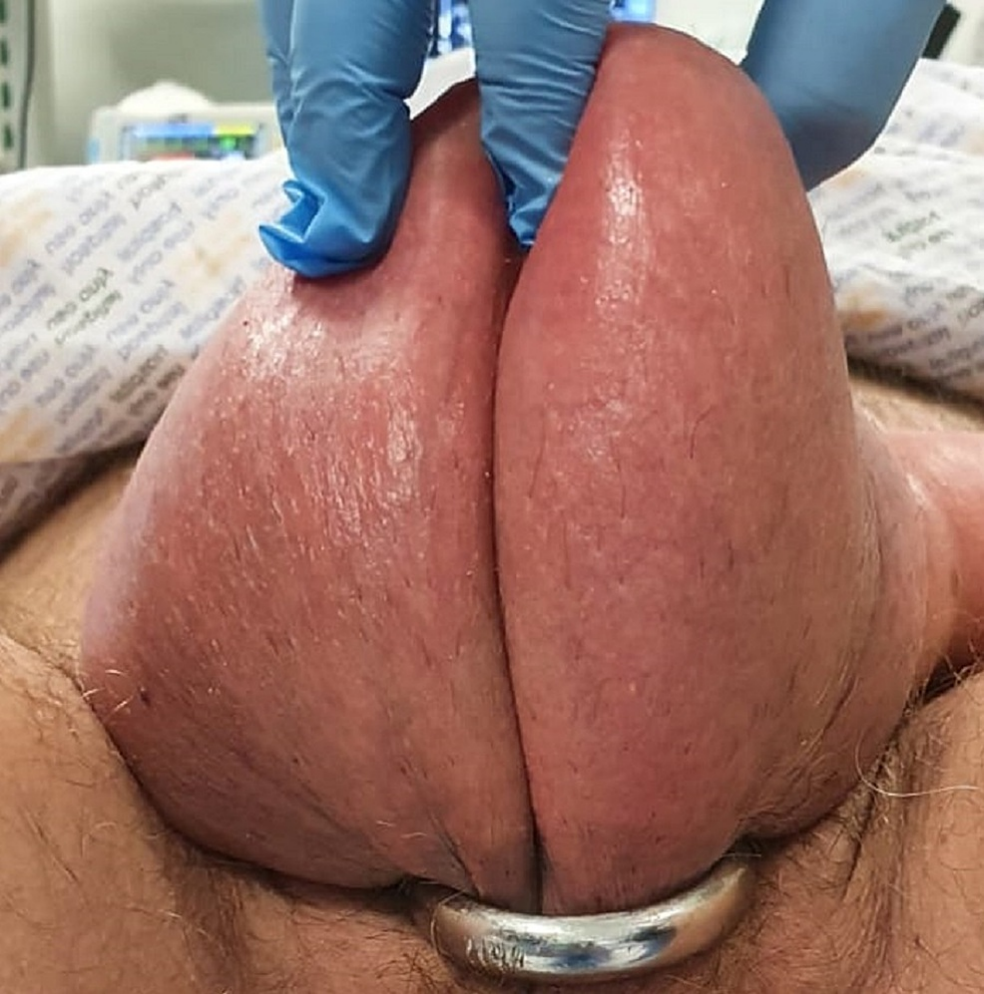
Penile ring entrapping both the penis and the scrotum (ventral view).

A 61-year-old male presented to the emergency ward with painful edematous swelling of the penis along with urine retention. The patient developed these symptoms after the insertion of a penile ring 24 hours back for enhancing penile erection and sexual performance. The patient failed to remove or cut the ring with either a metal saw or grinder. The patient had a history of ED for the last two years. On physical examination, it was revealed that the patient developed penile lacerations at the ring site along with ecchymosis and edema (Figures 1-3). In addition, he developed urinary retention secondary to the significant penoscrotal edema. The emergency team attempted to manually remove the penile ring. However, all the attempts were unsuccessful.

Therefore, the patient was transferred to the operative theatre to remove the penile ring under general anesthesia. Given the fact that the metal ring was very thick, several failed efforts lasted longer than five hours. The iron saw and metal wire were employed. In addition, orthopedic instruments were utilized without fruitful results.

**Figure 4:**
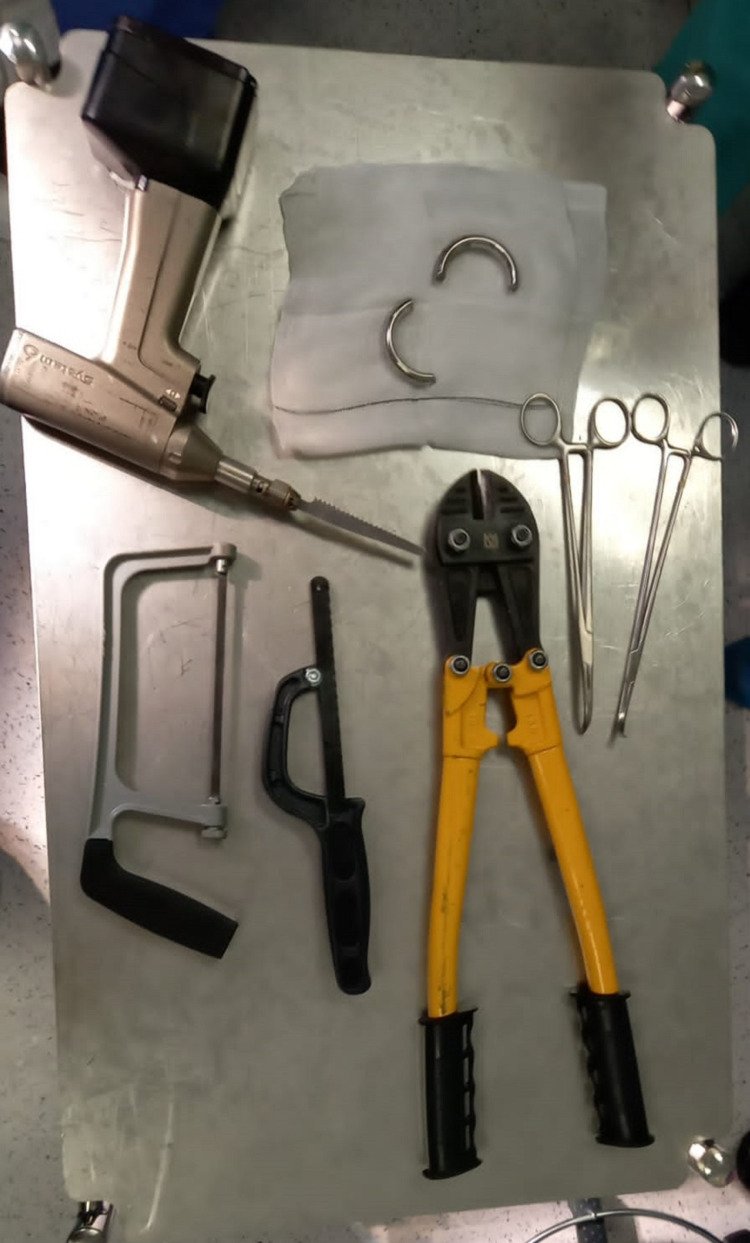
Successfully removed penile ring using a special iron cutter.

Eventually, the hospital maintenance team was summoned, and they used a special iron cutter to shatter the metal ring (Figure 4). To empty the bladder, a 12 Foley urethral catheter was inserted, and clear urine was observed. The catheter was taken out. The patient's recovery following anesthesia was uneventful, and he was discharged the same day on antibiotics and painkillers. The patient was referred to a sexual health clinic for counseling and management of his ED.

## Discussion

The use of metal rings to sustain a penile erection is unique, and the ring might become entrapped on the penis [5]. Because of its rarity, many practitioners are unfamiliar with this crisis. The care is difficult, and the surgeon frequently examines a variety of procedures to remove the ring from the inflamed penis. The purpose of this case report is to raise awareness of penile ring entrapment and to discuss frequent ways of extrication. Children occasionally tie elastic bands around male genitalia as part of childhood pranks or to avoid urinary incontinence, and removal by cutting is simple [6]. When a boy appears with unexplained penile enlargement, ulceration, and urine leakage, the doctor should keep this in mind [7,8]. The use of metal rings for sexual reasons in adults has been reported [1,5]. Complications can be avoided if the ring is presented and extracted as quickly as possible. After 72 hours, patients are more likely to experience significant complications such as pressure necrosis, urethral fistula, penile gangrene, and stricture [2].

There are no conventional procedures or guidelines for removing an entrapped penile ring, and it can be done with non-medical instruments. Some cases of penile ring entrapment are challenging to cure and need creativity and a multidisciplinary approach [9,10]. The string approach is straightforward, and it may be used to free a thin metal ring in the early stages of penile entrapment without penile edema. The string is threaded between the space between the ring and the penile skin. If there is significant edema, the tension of the string on the penile skin may cause lacerations [5]. Cutting is the most commonly used method of removal, despite the difficulty and danger of penile damage [5]. If a massive orthopedic cutter can be inserted beneath the ring to cut it, it will be the quickest but not always the safest technique of removing a thick metal ring. A little cutter, on the other hand, that can easily penetrate beneath the ring without harming the penis is unlikely to cut heavy metal. A hacksaw, Gigli saw, or angle grinder may potentially harm the penis due to insufficient protection in the tiny operating field and heat buildup [10,11]. Sprinkling ice-packed normal saline on the interphase will assist to minimize heat and avoid burns [12].

Aspiration decompression is an invasive procedure for draining blood from distended corpora. This shrinks the penis, allowing the ring to be removed [12]. Because this approach does not remove inflammatory edema from the penile skin and interstitial spaces, it may be coupled with other operations to accomplish extrication. The surgical degloving of the penile skin and Buck's fascia from the corpora is analogous to hypospadias surgeries. The flaps are decreased by passing them through the ring, which may then be easily slid distally over the degloved shaft [13]. However, the edematous glans cannot be degloved, which adds to the difficulty. This procedure necessitates advanced training, and problems such as wound disintegration, urethral damage, and flap necrosis are possible.

In our patient, under general anesthesia, we tried most of the procedures, and the hospital maintenance team was eventually summoned, and they used a special iron cutter to shatter the metal ring after the failure of all the above protocols.

## Conclusions

The use of a metal ring for the penis should be revaluated and scientific discussion should be made about the harms of its use. These types of rings are being used and applied in the absence of medical supervision. However, in case of overwhelming complications, the medical team would be responsible for dealing with the problem, which leads to more strain on the limited health system resources. This case might be an example of how to deal with the problem with a severe lack of guidance methods.
